# Genome-wide identification of the bHLH gene family in *Scutellaria baicalensis* and their relationship with baicalin biosynthesis under drought stress

**DOI:** 10.3389/fpls.2024.1506805

**Published:** 2025-01-27

**Authors:** Yingxin Sun, Beier Wang, Lichao Zhang, Xiaohan Zheng, Peng Xu, Meng Zhang, Meiguang Han, Peng Di, Mei Han, Lin Cheng, Limin Yang

**Affiliations:** ^1^ Cultivation Base of State Key Laboratory for Ecological Restoration and Ecosystem Management, College of Chinese Medicinal Materials, Jilin Agricultural University, Changchun, China; ^2^ Technology Service Center on Ecological Planting of Chinese Herbal Medicine in Chengde, Chengde, China

**Keywords:** *Scutellaria baicalensis*, bHLH gene family, expression pattern, baicalin biosynthesis, drought stress

## Abstract

The bHLH gene family plays a critical role in regulating internal responses in plants. Although the pharmacological properties of *Scutellaria baicalensis* have been extensively studied, its bHLH gene family remains poorly investigated. In this study, 142 *SbbHLH* genes were identified using the complete genome data of *S. baicalensis*. Phylogenetic and conserved motif analyses were performed. Gene duplication events were analyzed, and cis-element analysis was conducted to explore regulatory factors. The expression patterns of these genes in different tissues and under drought stress were investigated using transcriptome data and qRT-PCR analysis. Phylogenetic and conserved motif analyses revealed that the gene structures within each *SbbHLH* clade are relatively conserved. Gene duplication analysis identified 29 duplication events in the SbbHLH gene family, most of which involved gene pairs under purifying selection. Cis-element analysis revealed that these genes are regulated by various environmental and hormonal factors. Transcriptomic data and qRT-PCR results demonstrated tissue-specific expression patterns for the 142 *SbbHLH* genes. Additionally, *bHLH* genes potentially involved in baicalin biosynthesis were identified under drought stress. The findings suggest that under drought stress, *SbbHLH74, SbbHLH98*, and *SbbHLH142* are regulated by a network centered on *SbbHLH53*, which enhances baicalin biosynthesis. In conclusion, this study provides a comprehensive analysis of the bHLH gene family in *S. baicalensis* and identifies 4 potential *SbbHLH* genes involved in regulating baicalin biosynthesis under drought stress.

## 1 Introduction

Transcription factors (TFs) constitute a crucial class of regulatory factors in plants (Capella et al., 2015). As implied by their name, all bHLH gene families share a conserved bHLH domain (Birkenbihl et al., 2017). The bHLH domain consists of two key regions: the helix-loop-helix region and the basic region. This domain, widely distributed across eukaryotes (Lei et al., 2024). The recognition region of *bHLH* genes is located at the N-terminus, consisting of a basic region of 10-15 amino acids (aa) that can specifically recognize and bind to G-box and E-box motifs within target sequences (Toledo-Ortiz et al., 2003; Wenjing et al., 2016). In contrast, the amino acid sequence at the C-terminus is longer, spanning 40-50 amino acids, and forms two α-helical structures. The C-terminal region plays a regulatory role, such as enhancing protein interactions or facilitating the formation of protein complexes (Nair and Burley, 2000; Feller et al., 2011a; Huang et al., 2024). Different plant species possess varying numbers of genes within the bHLH gene family. For instance, 127 *bHLH* genes have been identified in *Salvia miltiorrhiza (* Zhang et al., 2015), 165 in *Oryza sativa* (Heim et al., 2003), 85 in *Ginkgo biloba* (Zhou et al., 2020), 161 in *Arabidopsis thaliana* (Li et al., 2006), 118 in *Juglans mandshurica* (Li et al., 2024), and 127 in *Panax ginseng* (Zhu et al., 2020). These publicly available research findings not only provide a solid foundation for subsequent gene family identification but also offer valuable original data for the functional and evolutionary analysis of these gene families.

Numerous studies have elucidated the fundamental functions of the bHLH gene family. In terms of growth and development, as well as in response to adverse stress, *bHLH* genes actively participate in metabolic regulation and signal transduction activation (Li et al., 2024). For instance, the first *bHLH* gene identified was isolated from *A. thaliana*, where it promotes the growth of roots, stems, leaves, and floral organs (Chen et al., 2024). Additionally, research has shown that the *GhDEL65* gene, a member of the bHLH gene family in *Gossypium hirsutum*, interacts with other genes to form a MYB-bHLH-WD40 complex, which regulates cotton fiber development (Shangguan et al., 2016). In addition, the bHLH gene family plays an important role in regulating plant responses to environmental stresses, including cold (Xue et al., 2022), mechanical damage (Zhang et al., 2023), elevated salinity (Su et al., 2023), and drought (Liang et al., 2023). Studies have shown that *OsbHLH148* in rice is activated by drought treatment. The *OsbHLH148* gene forms a complex with the *OsCOI1* and *OsJAZ1* genes to jointly regulate the response to drought stress (Seo et al., 2011). Similarly, the *MdCIB1* gene, a member of the bHLH gene family in *Malus domestica*, exhibits a similar function. Researchers used heterologous expression to transform the gene into *A. thaliana* and found that it accelerated root growth, resulting in enhanced drought resistance (Ren et al., 2021). Secondary metabolites are an integral part of plant metabolism, and the bHLH gene family also plays a key role in regulating the synthesis of secondary metabolites, such as alkaloids (Singh et al., 2021), terpenoids (Mertens et al., 2016; Mohammad et al., 2023), phenolic acids (Liu et al., 2022), and flavonoids (Jia et al., 2021; Li et al., 2022). For example, the upregulation of the *SmbHLH3* gene in *S. miltiorrhiza* can reduce the synthesis of phenolic acids. Further studies have shown that it regulates phenolic acid biosynthesis by forming a complex with *SmHPPR1* and *SmTAT1 (* Zhang et al., 2020). The biosynthesis of anthocyanins in *Vaccinium* spp. can be enhanced by upregulating the *VcbHLH1* gene (Zhao M. et al., 2019). Collectively, existing studies highlight the broad role of the bHLH gene family and emphasize the importance of further research on its members.

*Scutellaria baicalensis* has been used for over 2,000 years in traditional Chinese medicine to treat ailments related to the liver and lungs, characterized by its bitter and cold properties (Zhao et al., 2016; Zhao Q. et al., 2019; Geng et al., 2023). Modern pharmacological studies have shown that baicalin, the primary bioactive component of *S. baicalensis*, possesses beneficial effects, including anti-tumor, anti-viral, and analgesic properties (Wang et al., 2018). It also has potent inhibitory effects on various pathogens (Wang et al., 2022; Jia et al., 2023). The components of *S. baicalensis* that are responsible for treating diseases are root-specific flavonoids (RSFs), including baicalein, wogonin, baicalin, and wogonoside (Fang et al., 2023). Unlike typical 4-hydroxy flavonoids, such as scutellarein, they lack a hydroxyl group in their chemical structure, classifying them as 4’-deoxy RSFs (Pei et al., 2022). The biosynthetic pathway of RSFs begins with the conversion of phenylalanine to cinnamoyl-CoA by phenylalanine ammonialyase (PAL) and cinnamate-CoA ligase-like 7 (CLL-7). This intermediate is then condensed by a specific chalcone synthase (CHS-2) and isomerized by chalcone isomerase (CHI) to produce pinocembrin, a flavanone lacking a 4’-OH group (Zhao et al., 2016). Subsequently, chrysin is synthesized by flavone synthase II-2 (FNSII-2), while flavone 6-hydroxylase (F6H) converts it to baicalein. Alternatively, it can be acted upon by flavone 8-hydroxylase (F8H) and phenylpropanoid and flavonoid *O*-methyltransferases (PFOMT) to produce wogonin (Zhao et al., 2018; Zhao Q. et al., 2019). Finally, baicalin and wogonoside are produced through the glucuronidation of wogonin and baicalein by flavonoid 7-*O*-glucuronosyltransferases (UBGAT) (Nagashima et al., 2000). Notably, the baicalein content in *S. baicalensis* has been observed to increase under drought stress conditions, with moderate drought typically promoting the accumulation of flavonoids (Cheng et al., 2018). Additionally, transcription factor families play a significant role in regulating baicalin biosynthesis under drought conditions (Cheng et al., 2023). However, studies on bHLH transcription factors in *S. baicalensis* are still limited, and the molecular kinetics underlying flavonoid synthesis in this species remain poorly understood.

This study performed a comprehensive genome-wide analysis of the bHLH gene family in *S. baicalensis*. The analysis included the assessment of physicochemical properties, evolutionary relationships, gene structures, chromosomal locations, cis-regulatory elements, and synteny of the *bHLH* genes. Additionally, transcriptome data were analyzed to assess the tissue-specific expression patterns of *bHLH* genes and their correlation with flavonoid biosynthesis under drought stress. This approach aimed to elucidate the regulatory mechanisms underlying *bHLH* genes in *S. baicalensis*. The findings provide a foundational framework for exploring the biological functions of *bHLH* genes and offer deeper insights into the molecular regulatory mechanisms of *SbbHLH* genes.

## 2 Materials and methods

### 2.1 Plant materials and drought treatment

For drought treatment, two-year-old *S. baicalensis* plants were cultivated in 7.5 × 45 cm nutrient pots under controlled greenhouse conditions. The culture parameters were set to a 24-hour cycle with the following settings: from 0 to 8 hours, a temperature of 15°C, light intensity of 0 lux, and 50% humidity; from 8 to 24 hours, a temperature of 22°C, light intensity of 30,000 lux, and 50% humidity. We determined the most suitable time and the most suitable concentration of polyethylene glycol (PEG) solution through the experiments of different drought stress time and different drought stress degree in the early stage.The treatment group was irrigated with 120 mL of 20% PEG solution, while the control group received an equal volume of distilled water. Plants were harvested 24 hours after treatment. A total of nine samples were collected from each group, with every three samples pooled together. The pooled samples were wrapped in aluminum foil, snap-frozen in liquid nitrogen, and stored at -80°C. Each treatment was conducted with three biological replicates.

### 2.2 Identification and characterization of *SbbHLH* genes

*S. baicalensis* genome data (GWHAOTO00000000) were obtained from CNCB (https://www.cncb.ac.cn/, accessed on 8 April 2024) (Xu et al., 2020). To identify potential *bHLH* genes, two complementary approaches were employed. First, the Hidden Markov Model (HMM) for the bHLH domain (PF00010) was retrieved from the InterPro database (https://www.ebi.ac.uk/interpro/, accessed on 10 April 2024), and the *S. baicalensis* protein sequence database was scanned using HMMER 3.0 (Huang et al., 2024). Second, *A. thaliana* bHLH protein data were downloaded from the Plant Transcription Factor Database (PlantTFDB, https://planttfdb.gao-lab.org/, accessed on 10 April 2024) (Mohammad et al., 2023). BLAST 2.15.0 was performed using *A. thaliana* bHLH protein sequences as queries, with an e-value <1^e−5^, to identify potential bHLH protein sequences in *S. baicalensis*. Redundant protein IDs and sequences were removed, and all identified candidates were further verified using the Pfam (https://www.ebi.ac.uk/interpro/, accessed on 15 April 2024) (Mohammad et al., 2023), SMART (https://smart.embl.de/, accessed on 16 April 2024) (Huang et al., 2024), and Batch CD-search (https://www.ncbi.nlm.nih.gov/cdd/, accessed on 15 April 2024) databases. The physicochemical properties of SbbHLH proteins were analyzed using the ExPASy (https://web.expasy.org/protparam/, accessed on 6 May 2024) (Zhang et al., 2015). Subcellular localization of the SbbHLH proteins was predicted using multiple tools, including WoLF-PSORT (https://wolfpsort.hgc.jp/, accessed on 6 May 2024) (Zhou et al., 2020), CELLO v.2.5 (http://cello.life.nctu.edu.tw/, accessed on 18 November 2024) (Huang et al., 2024), and BUCSA (https://busca.biocomp.unibo.it/, accessed on 18 November 2024) (Liang et al., 2023).

### 2.3 Phylogenetic analysis, chromosomal localization and collinearity analysis of *SbbHLH* genes

The SbbHLH and AtbHLH protein sequences were aligned using MAFFT v7.520. A phylogenetic tree was then constructed using the Maximum Likelihood (ML) method with 1000 bootstrap replicates and visualized using iTOL (https://itol.embl.de/, accessed on 20 May 2024). The ka/ks ratios were calculated with ka/ks Calculator 2.0 to assess natural purifying selection among target gene pairs. The *SbbHLH* genes of *S. baicalensis* were mapped to nine chromosomes using the genome data. Additionally, the collinear relationships between chromosome pairs were analyzed using TBtools v2.025 (Chen et al., 2023).

### 2.4 Gene structure, conserved motifs and cis-elements analysis of *SbbHLH* genes

Gene structure annotation was obtained using the GFF3 file of the *S. baicalensis* genome, and visualized using TBtools v2.025 (Chen et al., 2023). Conserved motifs within the SbbHLH proteins were identified using the MEME (https://meme-suite.org/meme/ accessed on 8 June 2024). The 2kb region upstream of the start codon of the *SbbHLH* gene in the *S. baicalensis* genome was used as the promoter sequence and subjected to analysis in the PlantCARE database (https://bioinformatics.psb.ugent.be/webtools/plantcare/html/ accessed on 10 June 2024) to predict potential cis-elements.

### 2.5 Expression pattern analysis of *SbbHLH* genes in different tissues

The transcriptome data of *S. baicalensis* in different tissues were downloaded from public resources (https://ngdc.cncb.ac.cn/bioproject/browse/PRJCA009556 accessed on 30 April 2024) (Hu et al., 2023). The sequence reads were processed using FastQC 0.11.8 to remove adapters and low-quality sequences, then assembled using HISAT2 2.1.0. The expression patterns of *SbbHLH* genes were analyzed using normalized values expressed as Fragments Per Kilobase Million (FPKM). A heatmap was generated to visualize these expression patterns, with Z-score normalization applied to the data.

### 2.6 Determination of biochemical indexes and baicalin content

Accurately weigh 0.5 g of fresh *S. baicalensis* root samples. Following the instructions provided in the assay kits from the Nanjing Jiancheng Bioengineering Institute, measure the activities of superoxide dismutase (SOD, A001-1-1) and peroxidase (POD, A084-3-1), as well as the concentrations of proline (A107-1-1) and soluble protein (A045-2-2). To determine the baicalin content, The root tissues of *S. baicalensis*to a constant weight. A 0.3 g sample of the dried powder was then combined with 10 mL of 70% ethanol. Microwave extraction was performed at 80°C for 6 minutes using the MARS6 system (CEM, USA). Following extraction, filter the solution and dilute it to a final volume of 50 mL with 70% ethanol. Pass 2 mL of the diluted solution through a 0.22 μm filter membrane to prepare the sample for analysis. The baicalin content was determined using high-performance liquid chromatography (HPLC, Agilent 1100, USA) according to the elution protocol described by Han et al (Zhong-Ming et al., 2011). The concentration was calculated based on the standard curve equation: y = 2626.76x − 96.06 (R² = 0.9997). Each sample was measured in triplicate, and the average value was reported as the final result, with the standard deviation represented as the error bar.

### 2.7 Transcriptiomic analysis of *S. baicalensis* under drought stress

RNA was extracted from the roots of both control and treatment groups of *S. baicalensis*. To ensure RNA quality, the total RNA was assessed and quantified using the Qubit fluorescence quantifier and the Qsep400 high-throughput biological fragment analyzer. Breaks intact RNA into short RNA fragments. Then reverse transcribed to form cDNA. The cDNA was purified and Polymerase Chain Reaction (PCR) amplification to complete the construction of cDNA library. After sequencing, the raw data were obtained. The raw data were filtered using FastQC 0.23.2 and Trimmomatic 0.36.5, and assembled using HISAT2 2.1.0. Normalization of the number of mapped reads against transcript length facilitated the calculation of gene alignment using FeatureCounts 1.6.2. Changes in gene expression were determined based on FPKM values.

### 2.8 Protein interaction and qRT-PCR analysis

The protein interaction network analysis of the differentially expressed genes was performed using the STRING database (https://cn.string-db.org/, accessed on July 27, 2024). Homologous genes from *A. thaliana* were selected to construct the interaction network, with a prediction score > 0.7 and a p-value < 1^e-10^. To validate the transcriptome data, 10 *SbbHLH* genes were randomly selected from the transcriptome data of *S. baicalensis*. Primers for each *SbbHLH* gene were designed using Primer Premier 5.0 (**Supplementary Table S1**). Quantitative real-time PCR (qRT-PCR) was performed to analyze these genes, with 18S rRNA serving as the reference gene. The qRT-PCR reaction mixture consisted of 10 µL of SYBR Premix Ex Taq (RR820 Takara, China), 1 µL of each primer, 1 µL of cDNA template, and 7 µL of ddH_2_O, making a total volume of 20 µL. The PCR protocol started with a pre-denaturation step at 95°C for 30 s, followed by 40 cycles of denaturation at 95°C for 5 s, annealing at 56°C for 30 s, and extension at 72°C for 15 s. Finally, the 2^-ΔΔCt^ method was used to normalize the experimental results, with *18S rRNA* as the internal reference gene. To ensure the reliability of the data, each sample was repeated three times. Statistical analysis was performed using one-way analysis of variance (ANOVA), and different lowercase letters indicate significant differences (*p* < 0.05).

## 3 Results

### 3.1 Identification of *SbbHLH* genes in *S. baicalensis*

To identify *bHLH* genes on a genome-wide scale in *S. baicalensis*, we used the AtbHLH proteins as queries and applied the HMM model containing the bHLH domain to search the complete amino acid database of *S. baicalensis*. After removing redundant sequences, the candidate SbbHLH genes were screened using the SMART, Batch CD-search, and Pfam databases, resulting in the identification of 142 *SbbHLH* genes (**Supplementary Table S2**). Based on their chromosomal locations, the 142 *SbbHLH* genes were renamed sequentially (*SbbHLH1*-*SbbHLH142*) (**Supplementary Table S3**). Analysis of their physicochemical properties revealed that the amino acid (aa) lengths of the SbbHLH proteins ranged from 91 (SbbHLH97) to 912 (SbbHLH56), with molecular weights (MW) varying from 10,431.7 Da (SbbHLH97) to 99,345.31 Da (SbbHLH56). The isoelectric points (pI) of these proteins ranged from 4.76 (SbbHLH104) to 11.54 (SbbHLH134), with an average pI of 6.94. Notably, 90 SbbHLH proteins had pI values below 7, classifying them as acidic, while 52 proteins exhibited pI values above 7, indicating they are basic. Subcellular localization predictions showed that 134 *SbbHLH* genes were localized to the nucleus, while the remaining proteins were distributed across other cellular compartments: chloroplasts (*SbbHLH42*, *SbbHLH43*, *SbbHLH58*, *SbbHLH66*, *SbbHLH69*, *SbbHLH83*), extracellular space (*SbbHLH95*), and mitochondria (*SbbHLH52*).

### 3.2 Phylogenetic analysis of *SbbHLH* and *AtbHLH* genes

**Figure 1:**
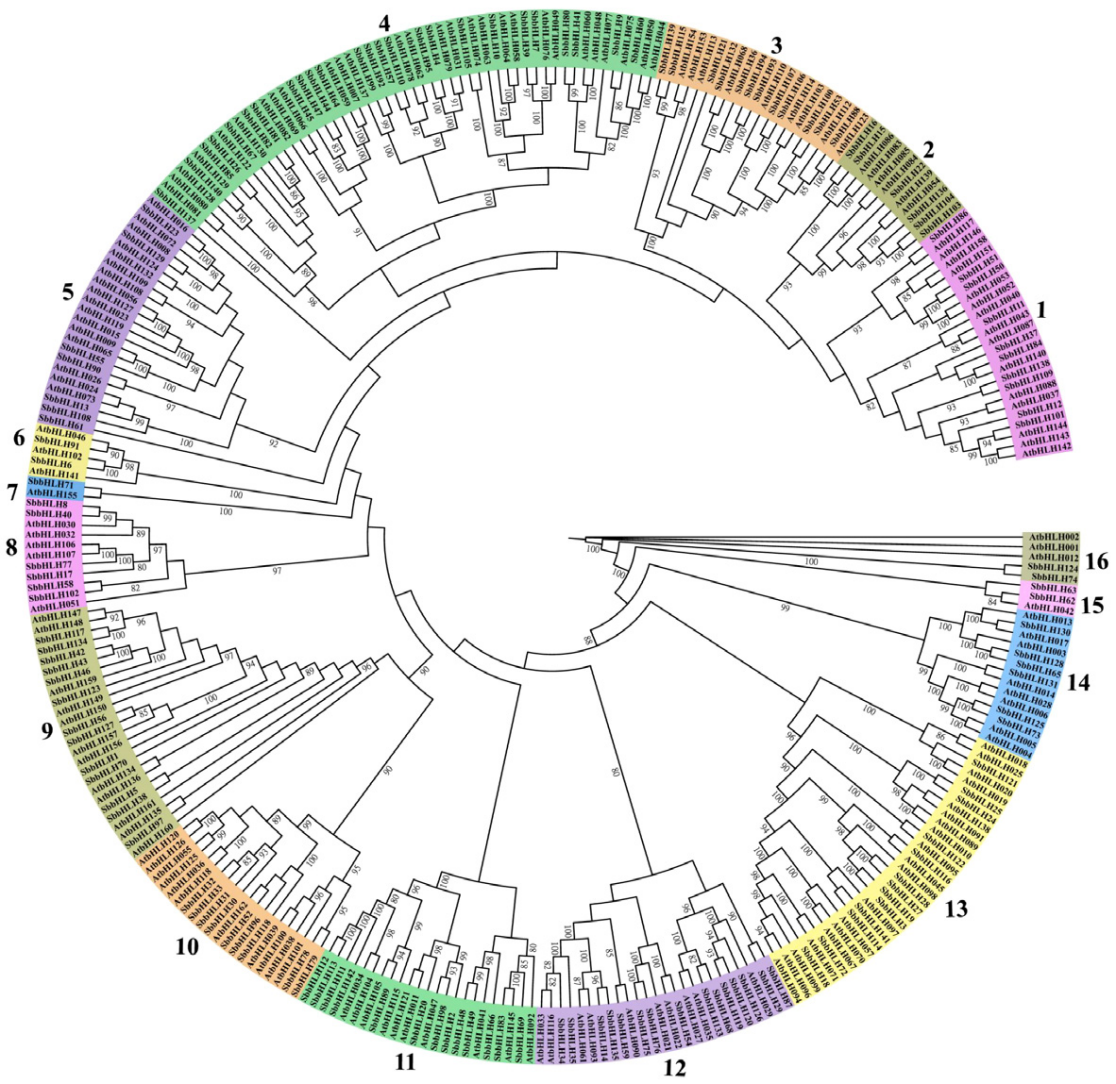
Phylogenetic tree of bHLH proteins in *S. baicalensis* and *A. thaliana*. Phylogenetic relationships were derived using the protein sequences by ML method with 1000 bootstrap. The bHLH proteins were grouped into 16 distinct branches, which are represented using various colors.

A phylogenetic tree was constructed using the protein sequences of 142 SbbHLH genes and 161 AtbHLH genes. The resulting phylogenetic tree was divided into 16 branches based on the homology of bHLH proteins (**Figure 1**). Significant variation was observed in the number of genes within each branch; branch 4 contained the highest number of members, with 25 *SbbHLH* genes (17.6%), while branch 7 had the fewest, comprising only one *SbbHLH* gene. Notably, each branch included *bHLH* genes from both *S. baicalensis* and *A. thaliana*, highlighting the evolutionary conservation of *bHLH* genes across species. Since genes within the same branch are likely to share similar functions, this phylogenetic analysis provides a foundational framework for predicting the potential functions of *SbbHLH* genes.

### 3.3 Gene structure and conserved motifs of *SbbHLH* genes

**Figure 2:**
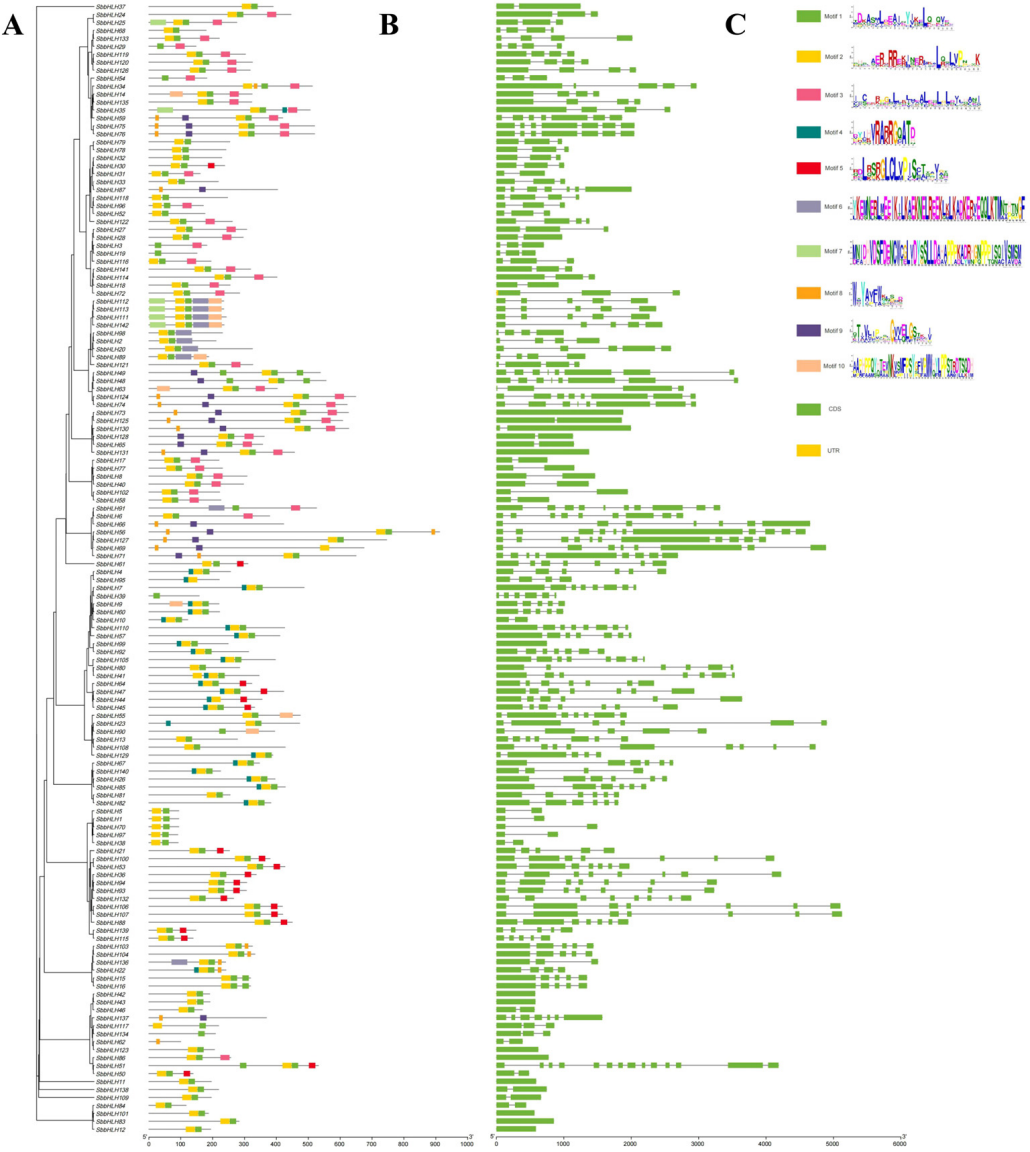
Conserved motifs and gene structure based on phylogenetic tree. **(A)** The phylogenetic tree of 142 *SbbHLH* genes. Phylogenetic relationships were derived using the protein sequences by ML method with 1000 bootstrap. **(B)** Conserved motifs were identified in *SbbHLH* genes. Different motifs are represented by different colors. **(C)** Schematic diagram of the exon and intron structures of 142 *SbbHLH* genes. The scale bar for gene sequence length is at the bottom right corner of each graph.

We constructed a phylogenetic tree to elucidate the relationships among all *SbbHLH* genes (**Figure 2A**). Based on the topology of the tree, we further analyzed conserved motifs and gene structures. A total of 10 conserved motifs were identified (**Figure 2B**). Notably, motifs 1 and 2 were present in all *SbbHLH* genes, with these two motifs constituting the complete bHLH domain. Specifically, motif 1 corresponds to the partial loop and helix 2 region of the bHLH domain, while motif 2 includes the basic region, helix 1, and part of the loop region. Similar motifs were typically distributed among genes within the same branch. For example, the branch containing *SbbHLH112*, *SbbHLH113*, *SbbHLH111*, and *SbbHLH142* displayed motifs 1, 2, 6, 7, and 10. Whereas the branch comprising *SbbHLH5*, *SbbHLH1*, *SbbHLH70*, *SbbHLH97*, and *SbbHLH138* only contained motifs 1 and 2. This distinct motif distribution suggests that these branches may have divergent functions, providing insights into the conservation and diversity of *SbbHLH* genes while supporting the phylogenetic tree classification. Gene structure analysis provides valuable insights into the phylogenetic relationships among *SbbHLH* genes. Among the 142 *SbbHLH* genes analyzed, the number of exons ranged from 1 to 14 (**Figure 2C**), with the majority containing between 2 and 8 exons. Notably, *SbbHLH51* exhibited the highest exon count at 14, while several genes, including *SbbHLH11*, *SbbHLH12*, *SbbHLH42*, *SbbHLH43*, *SbbHLH83*, *SbbHLH86*, *SbbHLH101*, and *SbbHLH123*, each contained only a single exon. Such variations likely reflect the unique functional roles and evolutionary trajectories of these genes, suggesting diverse regulatory mechanisms within the SbbHLH gene family. Moreover, the similarity in the number and length of exons within the same branch further supports the phylogenetic tree classification.

### 3.4 Chromosomal location, gene duplication and collinearity analysis of *SbbHLH* genes

**Figure 3:**
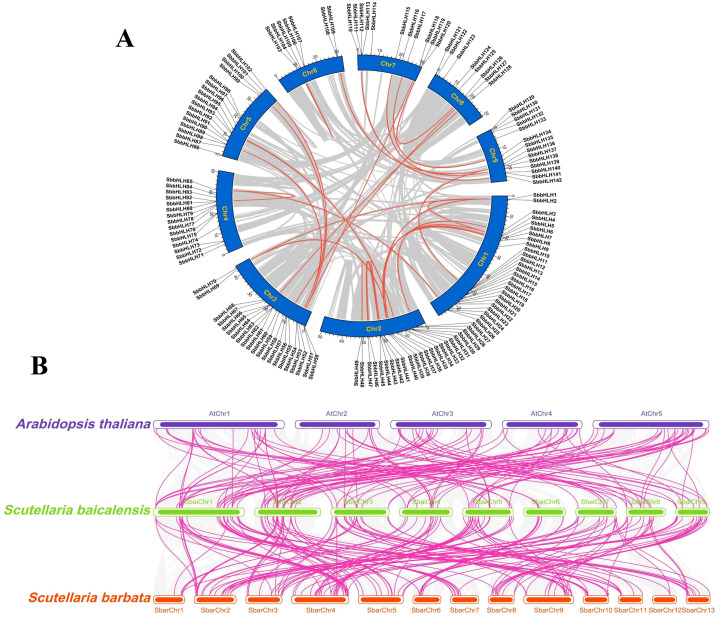
Chromosomal distribution and colinearity analysis of 142 *SbbHLH* genes. **(A)** Distribution of 142 *SbbHLH* genes on 9 chromosomes and collinearity analysis between chromosomes within the species. Chromosomes are represented by blue bars, and collinearity of gene pairs is indicated using red lines. **(B)** Colinearity analysis of *SbbHLH* genes among *S. baicalensis*, *S. barbata*, and *A. thaliana*. Collinear relationships of *bHLH* gene pairs are indicated using pink lines.

Chromosome localization analysis revealed that the 142 *SbbHLH* genes are distributed across all nine chromosomes of *S. baicalensis* (**Figure 3A**). Chromosome 1 contains the highest number of *SbbHLH* genes (26, 18.30%), while chromosome 6 has the fewest (7, 0.05%). Gene duplication events are known to facilitate the evolution and expansion of gene families in plants. To investigate the gene duplication events within the SbbHLH gene family, we analyzed a total of 142 *SbbHLH* genes (**Figure 3A**). Our analysis identified 26 segmental duplication events and 3 tandem duplication events. These findings were further supported by phylogenetic analysis, indicating the critical role of segmental duplication in the evolution of the SbbHLH gene family. Additionally, a ka/ks analysis was performed on 29 gene pairs (**Supplementary Table S4**). The results showed that, except for the *SbbHLH48/49* gene pair, which is under positive selection, the remaining 28 gene pairs underwent purifying selection. This further supports the notion that the SbbHLH gene family is crucial for environmental adaptation and the emergence of new functions. To clarify the phylogenetic relationships among bHLH gene families across different species, we constructed a collinearity map comparing the *bHLH* genes of *A. thaliana*, *S. barbata*, and *S. baicalensis* (**Figure 3B**). The analysis identified 122 homologous gene pairs between *S. baicalensis* and *A. thaliana* and 172 homologous gene pairs between the two Scutellaria species. The higher number of homologous gene pairs within Scutellaria indicates a closer evolutionary relationship between these species. Further analysis revealed that *S. baicalensis*, *A. thaliana*, and *S. barbata* had 121, 115, and 78 homologous genes, respectively. Notably, 70 homologous *bHLH* genes are shared among all three species, accounting for 49.30% of the total *SbbHLH* genes in *S. baicalensis*.

### 3.5 Prediction of cis-elements among *SbbHLH* genes

**Figure 4:**
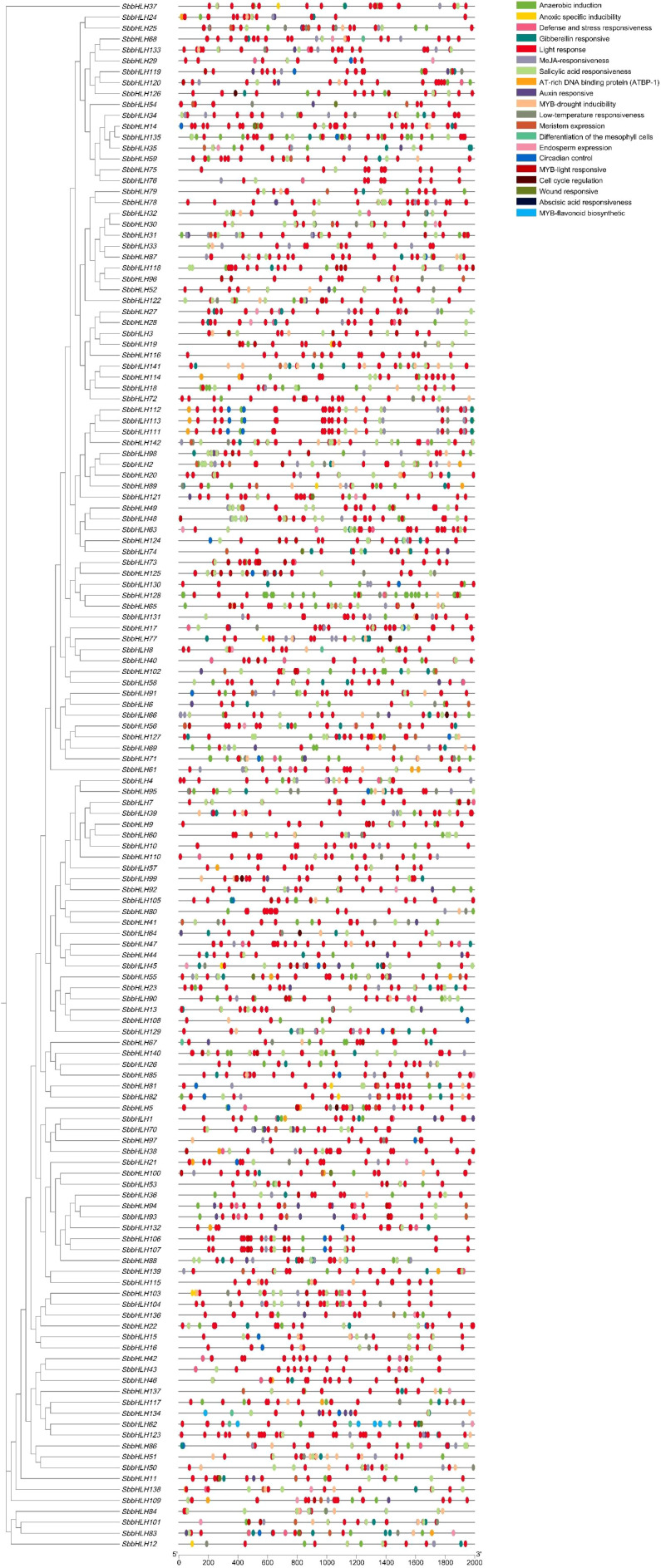
Analysis of cis-elements in the promoter region of 142 *SbbHLH* genes.

A total of 3,521 cis-elements were predicted across the 142 *SbbHLH* genes (**Figure 4**). These cis-elements were grouped into 21 types, which were further categorized into three main functional groups: growth and development, hormone response, and stress response. Among them, 2,081 cis-elements (59.10%) were associated with growth and development, followed by 910 (25.85%) linked to hormone responses and 530 (15.05%) related to stress responses. Notably, 138 of the 142 *SbbHLH* genes contained hormone-responsive elements, including those responsive to salicylic acid, abscisic acid, and gibberellins. This suggests that hormonal regulation plays a significant role in the expression of *SbbHLH* genes. Notably, although stress response elements constituted a smaller proportion overall, 139 *SbbHLH* genes were found to contain such elements, including those responsive to anaerobic conditions, low temperatures, and MYB-drought inducibility. Among them, *SbbHLH128*, *SbbHLH71*, and *SbbHLH135* had the largest number of stress response elements, with 30, 18, and 17 elements, respectively. Furthermore, seven *SbbHLH* genes were identified to contain MYB regulatory elements associated with flavonoid biosynthesis.

### 3.6 Expression patterns of *SbbHLH* genes in different tissues

**Figure 5:**
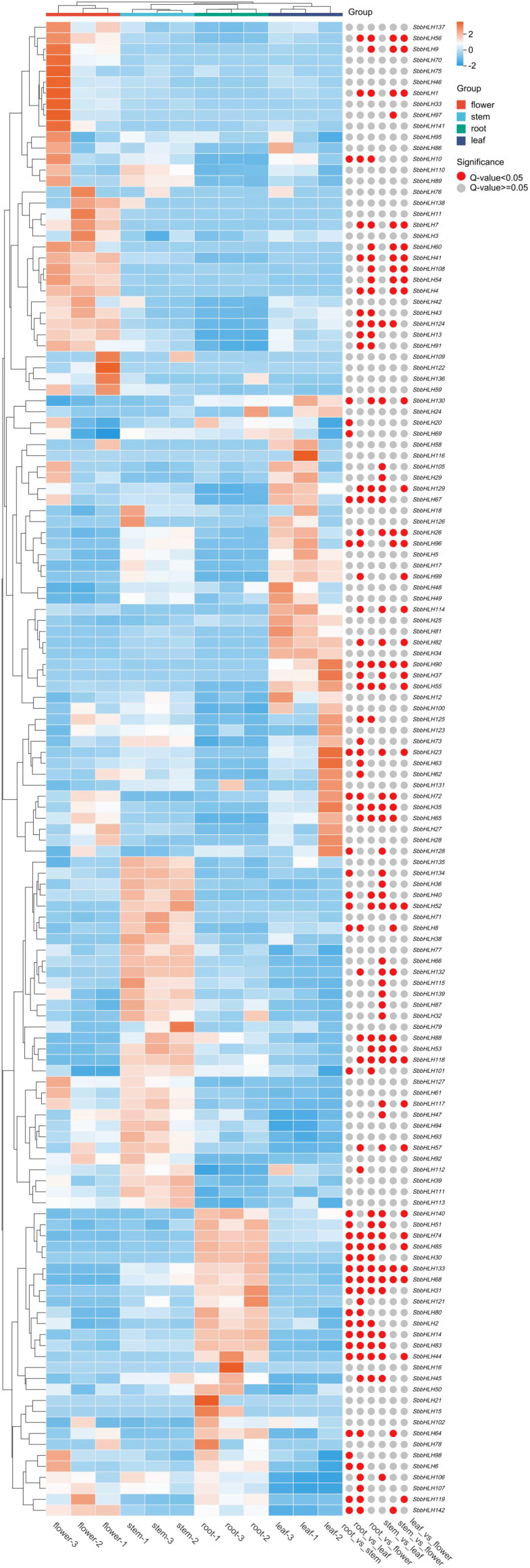
Expression patterns of *SbbHLH* genes in different tissues of *S. baicalensis*. Red circles indicate significant differences between the two comparison groups.

Baicalin is the primary medicinal component of *S. baicalensis* and classified as an RSF, and is predominantly synthesized in the root tissue. To explore the expression patterns of *SbbHLH* genes and identify potential genes involved in baicalin biosynthesis, we analyzed transcriptomic data from different tissues of *S. baicalensis*. Principal component analysis (PCA) revealed that the expression profiles of *SbbHLH* genes formed distinct clusters corresponding to the four tissue types (**Supplementary Figure S1**). This clustering was primarily driven by specific *SbbHLH* genes with high contributions to the principal components, indicating their tissue-specific expression patterns. Almost all *SbbHLH* genes were expressed in the four tissue types, with the exception of six genes (*SbbHLH19*, *SbbHLH22*, *SbbHLH84*, *SbbHLH103*, *SbbHLH104*, and *SbbHLH120*). We generated a heatmap using the FPKM values of the remaining 136 *SbbHLH* genes, which were not uniformly expressed across different tissues (**Figure 5**). Notably, the number of differentially expressed genes (DEGs) was highest in the flowers compared to the root, stem, and leaf tissues. In flower tissue, we identified 13 significant DEGs, of which 9 were upregulated and 4 were downregulated. In root tissue, we found 10 upregulated and 2 downregulated genes, totaling 12 DEGs. In stem tissue, 8 DEGs were observed, while only 2 DEGs were found in leaves. To validate our findings, we measured the expression levels of 10 *SbbHLH* genes in different tissues by qRT-PCR (**Supplementary Table S1**), which further confirmed the stability of the transcriptome data (**Supplementary Figure S2**).

### 3.7 Changes in the physiology and gene expression of *S. baicalensis* under drought stress

**Figure 6:**
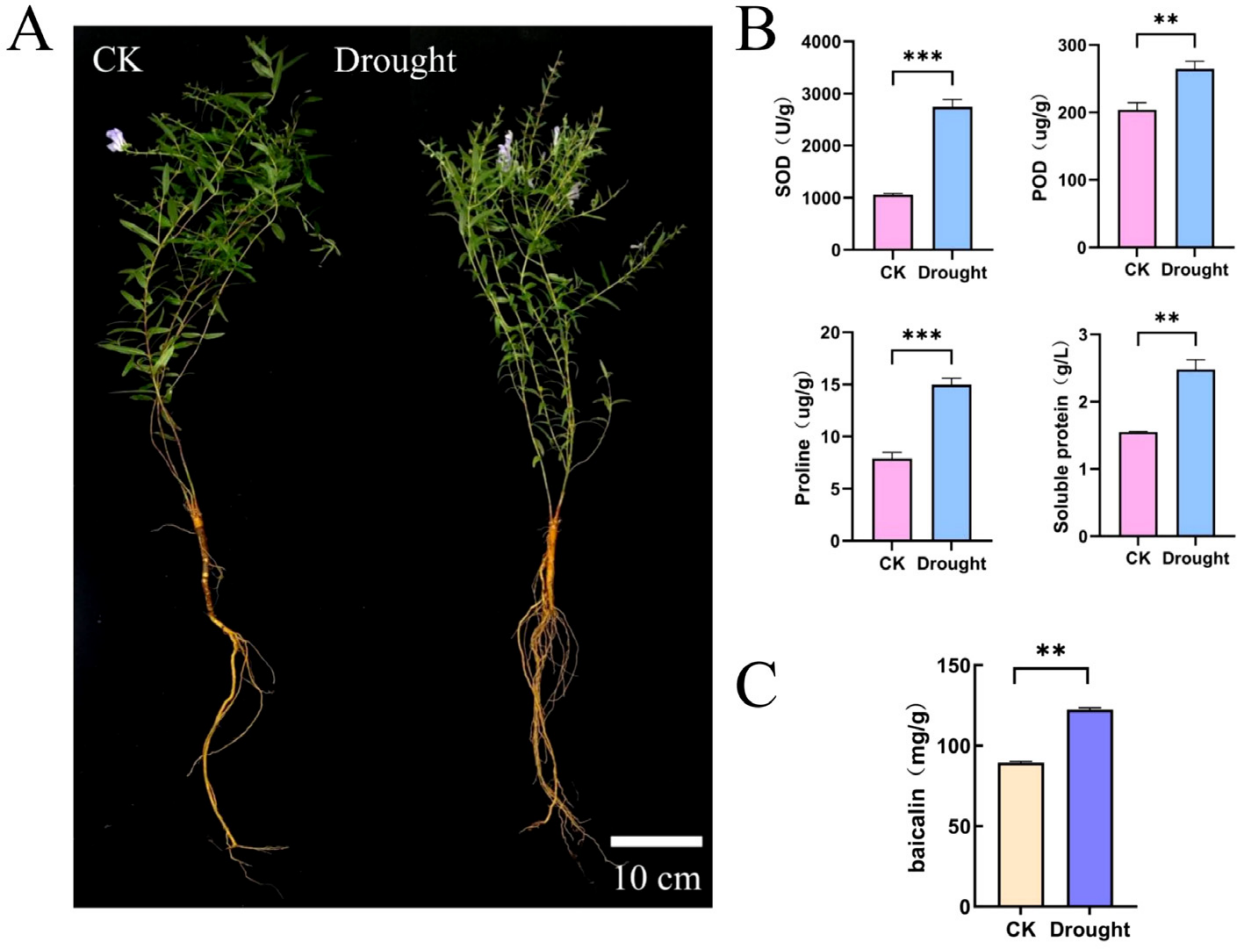
Physiological changes of *S. baicalensis* under drought stress. **(A)** The phenotypes of CK and drought treatment. Bar = 10 cm. **(B)** Protective enzyme activity and osmotic regulating substance content. **(C)** The baicalin content. Error bars were obtained from three measurements (Mean ± SD). Statistical analysis was conducted with one-way analysis of variance, ** and *** indicate *p* < 0.05 and *p* < 0.01.

**Figure 7:**
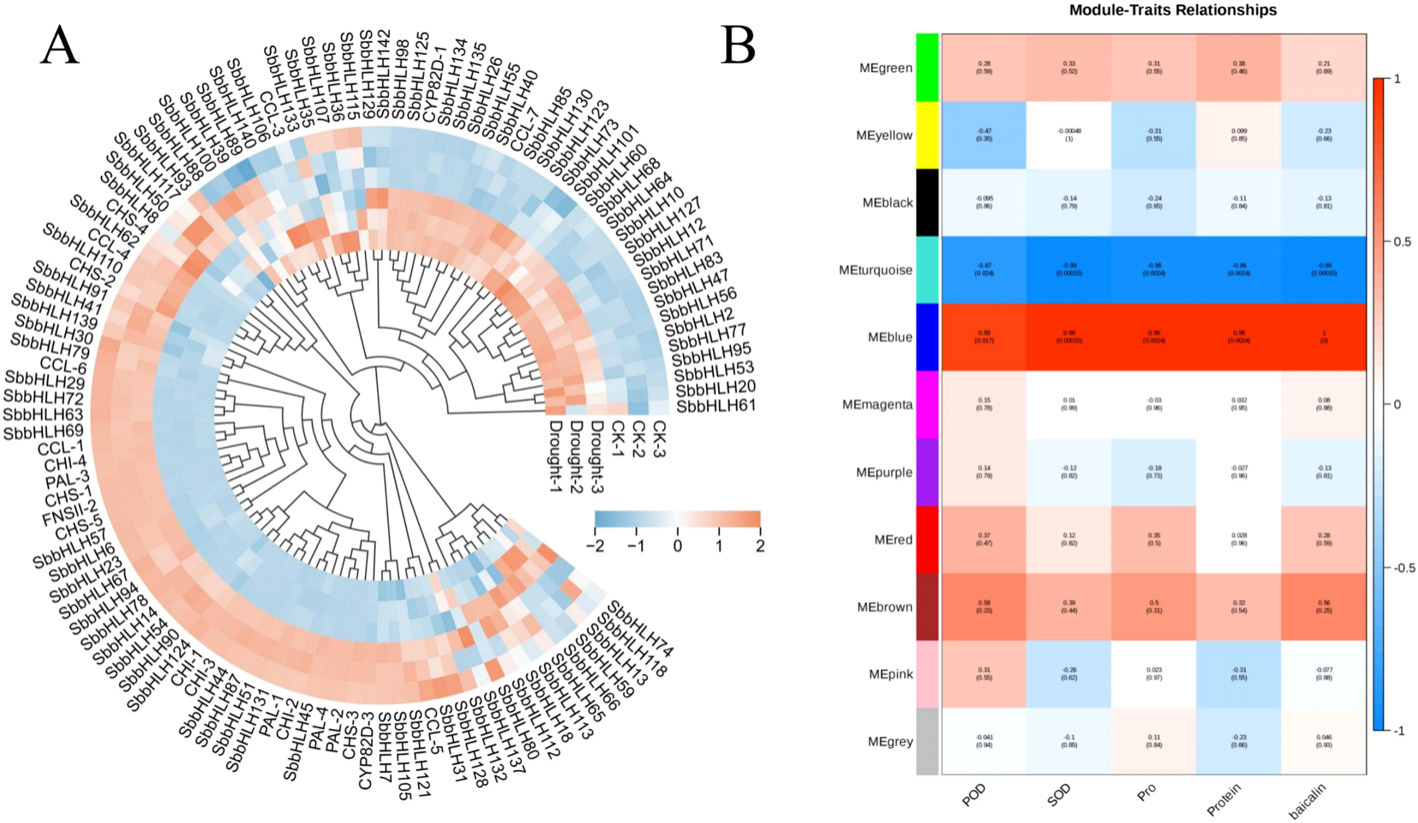
Cluster heatmap and WGCNA analysis of *S. baicalensis* under drought stress **(A)** Cluster heatmap of *SbbHLH* genes and baicalin biosynthesis genes in *S. baicalensis*. **(B)** Heatmap of the correlation between modules and physiological changes in *S. baicalensis*, with correlation coefficients and *p*-values in every graph.

To explore the effects of drought stress on the physiological and pharmacological components of *S. baicalensis*. we measured the activities of SOD and POD, as well as the contents of proline and Soluble Protein (**Figure 6A, B**). The results indicated that the activities of SOD and POD increased significantly under drought conditions, rising by 159.61% and 29.76%, respectively, compared to the control group. Similarly, the contents of proline and Soluble Protein also exhibited significant increases, with proline rising by 0.9-fold and Soluble Protein by 0.6-fold relative to the control group. Additionally, we assessed the changes in baicalin content under drought treatment. The results demonstrated a significant increase in baicalin content as well (**Figure 6C**). This increase in baicalin content is particularly beneficial for enhancing the quality of *S. baicalensis* as a medicinal material. We further analyzed the expression patterns of *SbbHLH* genes and baicalin biosynthesis genes under drought stress using transcriptomic data. A total of 82 *SbbHLH* genes and 22 baicalin biosynthesis genes were identified in both the control and drought-treated groups. After normalizing the FPKM values for these genes, a clustered heatmap was generated (**Figure 7A**). The results showed that the *CYP82D-1* and *CCL*-7 genes were upregulated under drought stress, along with 30 other *SbbHLH* genes exhibiting similar expression trends. This suggests that *CYP82D-1* and *CCL*-7, together with the 30 *SbbHLH* genes grouped into one class, may be involved in the biosynthesis of baicalin under drought stress. We divided the 15,179 genes into 11 distinct modules using weighted correlation network analysis (WGCNA) (**Figure 7B**). The turquoise module, containing 8,839 genes, is the largest, followed by the blue module with 4,981 genes. The purple module is the smallest, with only 75 genes. Notably, the expression pattern of genes in the blue module was strongly positively correlated with baicalin content, with correlations were both greater than 0.85 and *p*-values<0.01 (**Figure 7B**, **Supplementary Figure S3**). Therefore, we analyzed the genes in the blue module and searched for matches based on the original IDs of the 142 *SbbHLH* genes we identified. Among the blue module genes, we found 32 *SbbHLH* genes. Additionally, sequence alignment revealed three genes involved in baicalin biosynthesis (**Supplementary Table S5**). To validate the expression patterns of these genes, we performed qRT-PCR on 10 *SbbHLH* genes, which confirmed the reliability of the transcriptome data (**Supplementary Figure S4**).

### 3.8 Prediction of *SbbHLH* gene regulating baicalin biosynthesis under drought stress

**Figure 8:**
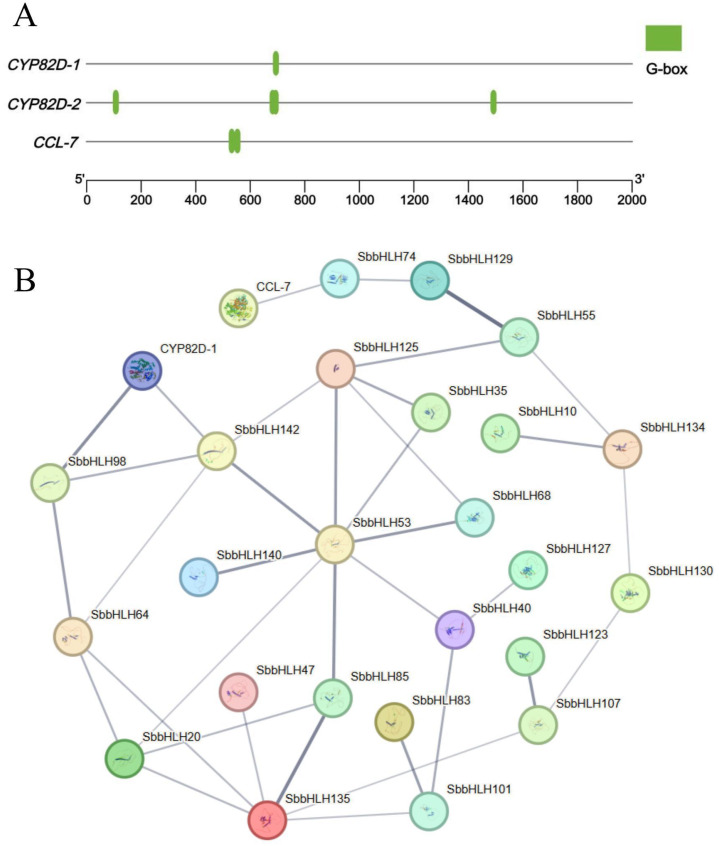
Analysis of cis-elements and protein interaction network. **(A)** Predicted cis-elements from 2 kb upstream of three key enzyme genes in the baicalin biosynthesis pathway. **(B)** Protein interaction network analysis. line thickness indicates the strength of data support.

Baicalin is one of the primary medicinal components of *S. baicalensis*. Our previous research demonstrated that moderate drought stress activates the stress response in *S. baicalensis*, inducing physiological changes that enhance baicalin content (Cheng et al., 2018; Cheng et al., 2023). We analyzed the promoter regions of *CYP82D*-2, *CYP82D*-1, and *CCL*-7, which are involved in the biosynthesis of the major secondary metabolite baicalin within the blue module. Our analysis revealed that the promoter regions of these genes in the baicalin biosynthetic pathway contain cis-elements associated with *bHLH* transcription factors (**Figure 8A**), suggesting that *CYP82D-2*, *CYP82D-1*, and *CCL-7* may be regulated by *SbbHLH* genes. We merged the 30 *SbbHLH* genes with expression patterns similar to *CYP82D*-1 and *CCL*-7 obtained from the clustered heatmap with the 32 *SbbHLH* genes from the blue module. After removing duplicates, a total of 36 unique *SbbHLH* genes were identified. Subsequently, we constructed a protein interaction network using the 36 *SbbHLH* genes and 3 baicalin biosynthesis pathway genes (**Supplementary Table S6**; **Figure 8B**; **Supplementary Figure S5**). The results showed that the *CYP82D-1* interacts with *SbbHLH142* and *SbbHLH98*, while *CCL-7* interacts with *SbbHLH74*. Notably, *SbbHLH53* emerged as a central node in the protein interaction network, interacting with multiple proteins, including *SbbHLH142*, *SbbHLH140*, *SbbHLH20*, *SbbHLH85*, *SbbHLH40*, *SbbHLH68*, *SbbHLH35*, and *SbbHLH125*. Phylogenetic analysis revealed that *SbbHLH53* in *S. baicalensis* shares a high degree of homology with *AtbHLH112* in *A. thaliana*. Previous studies have shown that, under drought conditions, *AtbHLH112* in *A. thaliana* is induced by drought stress and then regulates the expression of genes related to SOD, POD and proline metabolism. Based on previous studies and the findings of this study, we propose that the *SbbHLH* gene regulates the baicalin biosynthetic pathway under drought stress. Specifically, when *S. baicalensis* is subjected to drought stress, its physiological state undergoes significant changes, including alterations in SOD activity, POD activity, and proline content. At the genetic level, the expression of *SbbHLH53* is upregulated under drought stress, activating a regulatory network centered on this transcription factor and ultimately promoting baicalin biosynthesis. Within this complex regulatory framework, *SbbHLH74* may positively regulate the expression of *CCL-7*. Meanwhile, *SbbHLH98* and *SbbHLH142* are likely to enhance the expression of *CYP82D-1* (*F6H*), which accelerates the enzymatic conversion of chrysin to baicalein through the action of F6H. Ultimately, these interactions and regulatory mechanisms contribute to an increased biosynthesis rate of baicalin under drought stress conditions.

## 4 Discussion

### 4.1 Systematic identification and comprehensive analysis of *SbbHLH* genes in *S. baicalensis*

The bHLH gene family play crucial roles in signal transduction networks (Seo et al., 2011; Zhang et al., 2020; Yin et al., 2023). The identification of transcription factor families has been facilitated by the rapid increase in plant genomic data. The *bHLH* genes had been comprehensively analyzed in multiple species, including *Salvia miltiorrhiza (* Zhang et al., 2015), *Ginkgo biloba (* Zhou et al., 2020), *A. thaliana (* Li et al., 2006), *Juglans mandshurica (* Li et al., 2024), *Passiflora edulis (* Liang et al., 2023) and *Citrus sinensis (* Huang et al., 2024), underscoring the significance of this transcription factor family. However, comprehensive information regarding bHLH gene family in *S. baicalensis* remains scarce. Therefore, we identified and analyzed 142 *SbbHLH* genes in *S. baicalensis*. This number is comparable to that reported in *P. edulis* (138) (Liang et al., 2023) and *C. sinensis* (135) (Huang et al., 2024), fewer than in *A. thaliana* (161), and more than in *G. biloba (* Zhou et al., 2020) and *S. miltiorrhiza (* Zhang et al., 2015). The genome size and chromosome number of different species may lead to differences in the number of *bHLH* genes (Flagel and Wendel, 2009; Kamran et al., 2023).

The phylogenetic tree, comprising the 142 *SbbHLH* genes and 161 *AtbHLH* genes, showed that each *SbbHLH* gene clustered with at least one *AtbHLH* gene, forming sister branches (**Figure 1**). This clustering suggests that *bHLH* genes diversified prior to species evolution, thereby enhancing the reliability of classification and functional predictions for *SbbHLH* genes in *S. baicalensis (* Yao et al., 2024). The analysis of the conserved motifs in the 142 *SbbHLH* genes revealed that genes with similar conserved motifs are generally grouped within the same branch of the phylogenetic tree. This suggests that members within the same branch may share a common ancestor and perform similar functions, further supporting the phylogenetic relationships within *S. baicalensis* (**Figure 2A**). All *SbbHLH* genes contained motifs 1 and 2, indicating that these motifs represent the conserved domains of *bHLH* genes, forming the DNA-binding domain and the protein dimerization region (Kamran et al., 2023). In contrast, However, the distribution of motifs 2-10 was random but conserved within individual branches, reflecting the specific functions of genes within those branches. Among the 142 *SbbHLH* genes, the highest number of exons observed was 14, while 134 genes have more than two exons (**Figure 2B**). These results suggest that exon insertions or deletions may have occurred during evolution, contributing to the expansion of the SbbHLH gene family (Li et al., 2024).

The 142 *SbbHLH* genes are unevenly distributed across the nine chromosomes of *S. baicalensis* (**Figure 3A**), suggesting their involvement in evolutionary development (Reams and Neidle, 2004). Gene duplication events are common in transcription factor families, enabling rapid expansion and contraction in response to environmental changes. This process contributes to the quantitative variation of transcription factor families and promotes genetic diversity (Guo et al., 2024). Gene duplications are typically categorized into segmental and tandem duplications, with segmental duplications being particularly prevalent in plant transcription factor families (Yao et al., 2024). As anticipated, we identified three tandemly duplicated and 26 segmentally duplicated gene pairs in *S. baicalensis*, suggesting that segmental duplication likely plays a pivotal role in the evolution of *bHLH* genes and the development of novel functions for environmental adaptation (**Figure 3A**). Similar conclusions in other species, such as *J. mandshurica* and *C. praecox*, further support the idea that segmental duplication is a common mechanism driving the expansion of bHLH gene families (Kamran et al., 2023; Li et al., 2024). Collinearity analysis provided further insights into the origin and evolution of the bHLH gene family (**Figure 3B**). We identified 122 gene pairs between *S. baicalensis* and *A. thaliana*, and 172 gene pairs between Scutellaria species. This indicates a closer phylogenetic relationship within Scutellaria, while still showing 48.45% similarity with *A. thaliana*. This suggests that *bHLH* gene diversity may have emerged early in terrestrial plants. The ka/ks analysis revealed that *SbbHLH* genes primarily underwent purifying selection, reflecting highly conserved evolution (**Supplementary Table S4**) (Zhan et al., 2023). Additionally, we identified 70 collinear genes among *S. baicalensis*, *S. barbata*, and *A. thaliana*, which are crucial for understanding evolutionary dynamics.

### 4.2 The *SbbHLH* genes in *S. baicalensis* may regulate the biosynthesis of baicalin under drought stress

The growth and development of plant are complex processes influenced by both external environmental factors and internal hormone levels (Li et al., 2024). To further clarify the role of *SbbHLH* genes in *S. baicalensis*, we identified the cis-elements of 142 *SbbHLH* genes. The results showed that *SbbHLH* genes play significant roles in stress tolerance, hormone responses, and growth and development in *S. baicalensis* (**Figure 4**). Changes in transcription levels are a primary mode of regulation in organisms. We further examined the expression patterns of these 142 *SbbHLH* genes in different tissues (**Figure 5**). Our findings revealed that 136 *SbbHLH* genes were expressed in at least one tissue, with varying expression levels among different tissues, indicating tissue-specific expression of *SbbHLH* genes. Notably, 12 genes were highly expressed in the roots, suggesting their potential roles in root growth and development (Zhao et al., 2016; Cheng et al., 2018).

Given the importance of *S. baicalensis* for its antibacterial and anti-inflammatory properties (Wang et al., 2022). Moreover, the yield of *S. baicalensis* and the content of baicalin are closely linked to its market price, making the understanding of baicalin biosynthesis mechanisms a current research hotspot. Our study demonstrated that appropriate drought treatment could stimulate the production of secondary metabolites in *S. baicalensis*, including baicalin and baicalein (Cheng et al., 2018; Cheng et al., 2023). Under drought treatment, the physiological state of *S. baicalensis* undergoes significant changes, including the active removal of harmful substances through increased activity of protective enzyme, and osmotic adjustment via elevated proline and soluble protein content (**Figure 6B**). The content of the secondary metabolite baicalin also significantly increased, indicating that secondary metabolites help resist drought stress (**Figure 6C**). A substantial body of literature shows that these processes involve complex signal transduction between the environment, hormones, and transcription factors, ultimately altering the activity of downstream key enzymes and thereby affecting the biosynthesis of plant secondary metabolites (Guo et al., 2024). The bHLH gene family plays a crucial role in plant stress resistance. For example, overexpression of *MdCIB1* (cryptochrome-interacting *bHLH1*) in *Malus domestica* callus enhanced drought tolerance to PEG6000, while *MdCIB1* transgenic *A. thaliana* exhibited improved root development under drought stress (Ren et al., 2021; Li et al., 2024).

To investigate the potential roles of *SbbHLH* genes in regulating baicalin biosynthesis under drought treatment, we performed transcriptome data and qRT-PCR analysis on root tissues of *S. baicalensis* subjected to drought treatment and control treatments. To investigate the potential role of *SbbHLH* genes in regulating baicalin biosynthesis under drought stress, we performed transcriptome and qRT-PCR analyses on root tissues of *S. baicalensis* subjected to drought stress and control treatments. A total of 36 *SbbHLH* genes exhibited expression patterns consistent with the trend in baicalin content (**Figure 7A, B**). Additionally, we identified cis-elements associated with drought stress and hormone responses in the promoter regions of *SbbHLH* genes, further supporting their role in regulating the stress response in *S. baicalensis* (**Figure 4**). Protein interaction analysis suggested that *SbbHLH53* may act as a central component of the regulatory network under drought stress, modulating the expression of downstream genes, including *CCL7* and *CYP82D-1*, in response to drought conditions and hormonal changes (**Figure 8B**, **Supplementary Figure S5**). This suggests that these *bHLH* genes are part of a complex cross-regulatory network involving both drought stress and hormone induction. For instance, *ScbHLH5* and *ScbHLH65* exhibited significant positive correlations under various hormonal inductions in *Secale cereale*, indicating a synergistic regulatory effect between different endogenous hormonal metabolic pathways (Chen et al., 2024). Given the conservation of the bHLH gene family, we can predict the functions of *SbbHLH* genes through homology analysis (Feller et al., 2011b). Many *SbbHLH* genes have been functionally validated in *A. thaliana*, allowing us to infer their roles in *S. baicalensis*. The homologous gene of *SbbHLH53* in *S. baicalensis* is *AtbHLH112*, which has been shown to mediate physiological responses at the transcriptional level, including proline biosynthesis and ROS scavenging, thereby enhancing the salt and drought tolerance of *A. thaliana (* Liu et al., 2015).

The *bHLH* genes may exhibit complex regulatory mechanisms in response to abiotic stress and function by forming both homologous and heterologous complexes (Guo et al., 2024). Previous studies have indicated that the HLH domain facilitates dimer formation, with specific amino acid residues in the helix regions being essential for this process (Carretero-Paulet et al., 2010). Since the biosynthesis pathways of anthocyanins and baicalin share similarities, it is plausible that the *SbbHLH* gene may also form complexes through protein interactions, playing a crucial role in regulating downstream gene expression in response to drought stress signals. Our study revealed that 36 *SbbHLH* genes exhibited elevated expression under drought stress. Notably, we identified binding sites for *bHLH* genes in the promoter regions of key genes involved in baicalin biosynthesis (**Figure 8A**). The biosynthesis of baicalin may be influenced by drought stress, which can increase the expression of *SbbHLH* genes and consequently the content of baicalin (Yang et al., 2022; Cheng et al., 2023). Based on our analysis and previous studies, we hypothesize that *SbbHLH74*, *SbbHLH98*, and *SbbHLH142* play crucial roles in regulating baicalin biosynthesis. Specifically, under drought stress, the regulatory network centered on the *SbbHLH53* gene is activated, influencing the expression of *SbbHLH74*, *SbbHLH98*, and *SbbHLH142*, which in turn enhances the activities of CYP82D-1 and CCL-5 and promotes baicalin biosynthesis. Our study aims to further investigate the target genes that regulate baicalin biosynthesis to clarify the role of *SbbHLH* genes in this process. In future studies, we will validate the functions of *SbbHLH* genes and their regulatory mechanisms under drought stress.

## 5 Conclusions

This study represents the first genome-wide analysis of the *SbbHLH* gene family in *S. baicalensis*. We identified a total of 142 *SbbHLH* genes within the *S. baicalensis* genome, each corresponding to at least one homologous *AtbHLH* gene from *A. thaliana*. Conserved motif analysis indicated that *SbbHLH* genes are relatively conserved across different branches, further supporting their phylogenetic relationships. Chromosome localization revealed that the 142 *SbbHLH* genes are unevenly distributed across nine chromosomes. Segmental duplication was identified as the primary mechanism driving the amplification of *SbbHLH* genes, with 29 pairs of gene duplication events observed. We investigated the expression of *SbbHLH* genes in response to drought stress and explored their specific roles in regulating baicalin biosynthesis. The results showed that *SbbHLH* genes are interconnected and exhibit complex regulatory mechanisms under drought stress. Notably, *SbbHLH53* may serve as a core component of the regulatory network under drought conditions, while *SbbHLH74*, *SbbHLH98*, and *SbbHLH142* are potential candidate genes involved in the regulation of baicalin biosynthesis. In summary, our results provide a comprehensive analysis of the *SbbHLH* genes in *S. baicalensis* and providing new insights into the regulation of baicalin biosynthesis by *SbbHLH* genes under drought stress.

## Data Availability

The original contributions presented in the study are included in the article/**Supplementary Material**, further inquiries can be directed to the corresponding author/s.
