# Supplementary material for: Genome-wide identification of the bHLH gene family in Scutellaria baicalensis and their relationship with baicalin biosynthesis under drought stress
Source: Front Plant Sci. 2025 Jan 27;15:1506805. doi: 10.3389/fpls.2024.1506805 (PMC11807981; doi:10.3389/fpls.2024.1506805)
Supplement: Supplementary file 1 [file Table1.docx]

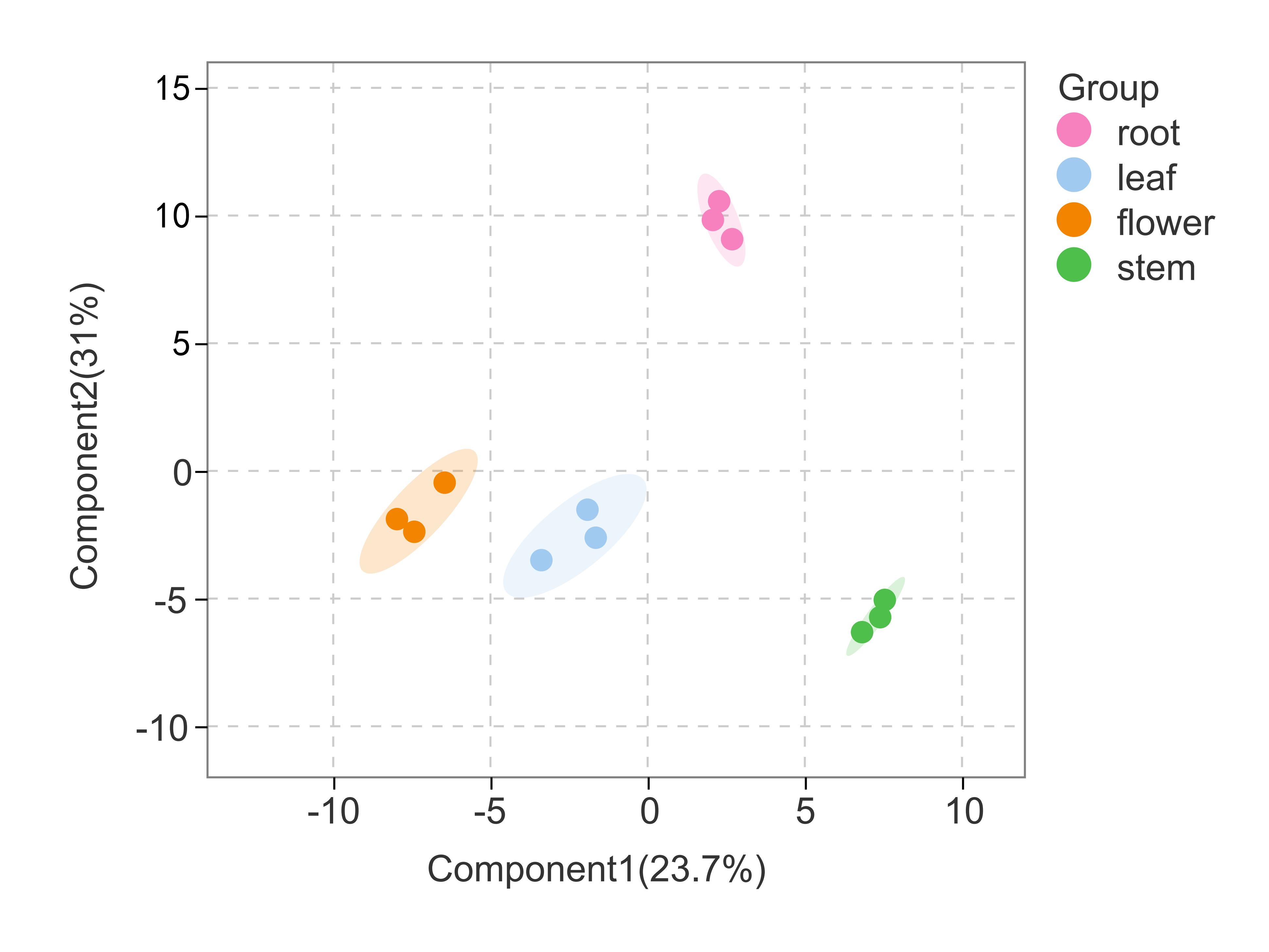


Fig. S1 PCA analysis of SbbHLH gene expression patterns in root, stem, leaf and flower tissues of *S. baicalensis*


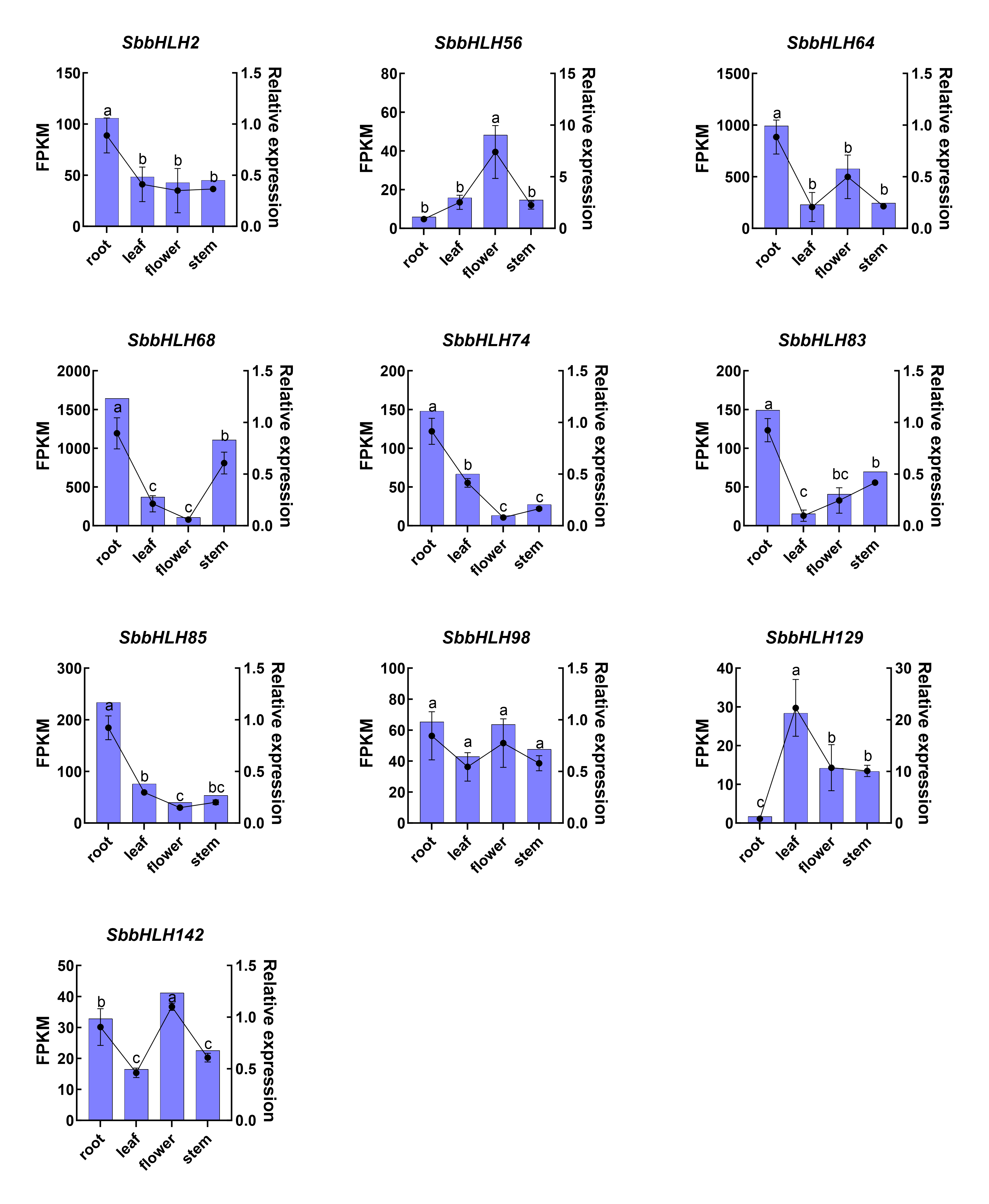


Fig. S2 Verification of transcriptome data of *S. baicalensis* root, stem, leaf and flower tissues. The blue bar graph is the FPKM value of the transcriptome data, and the black line graph is the relative expression level of qRT-PCR, repeated 3 times. Statistical analysis was conducted with one-way analysis of variance, different lowercase letters indicate significant differences (*p*<0.05).


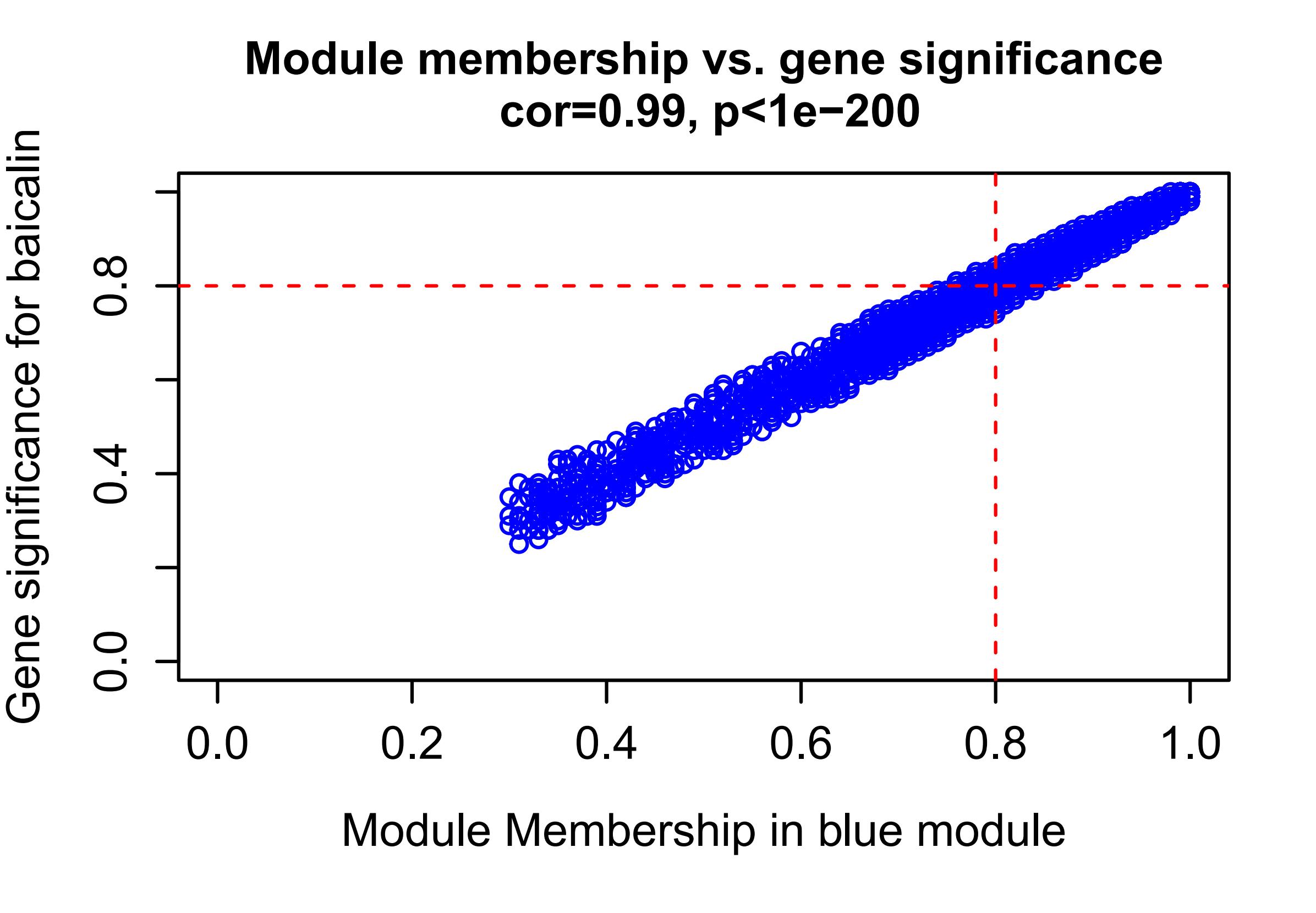


Fig. S3 Scatter plot of genes in the blue module identified by WGCNA analysis


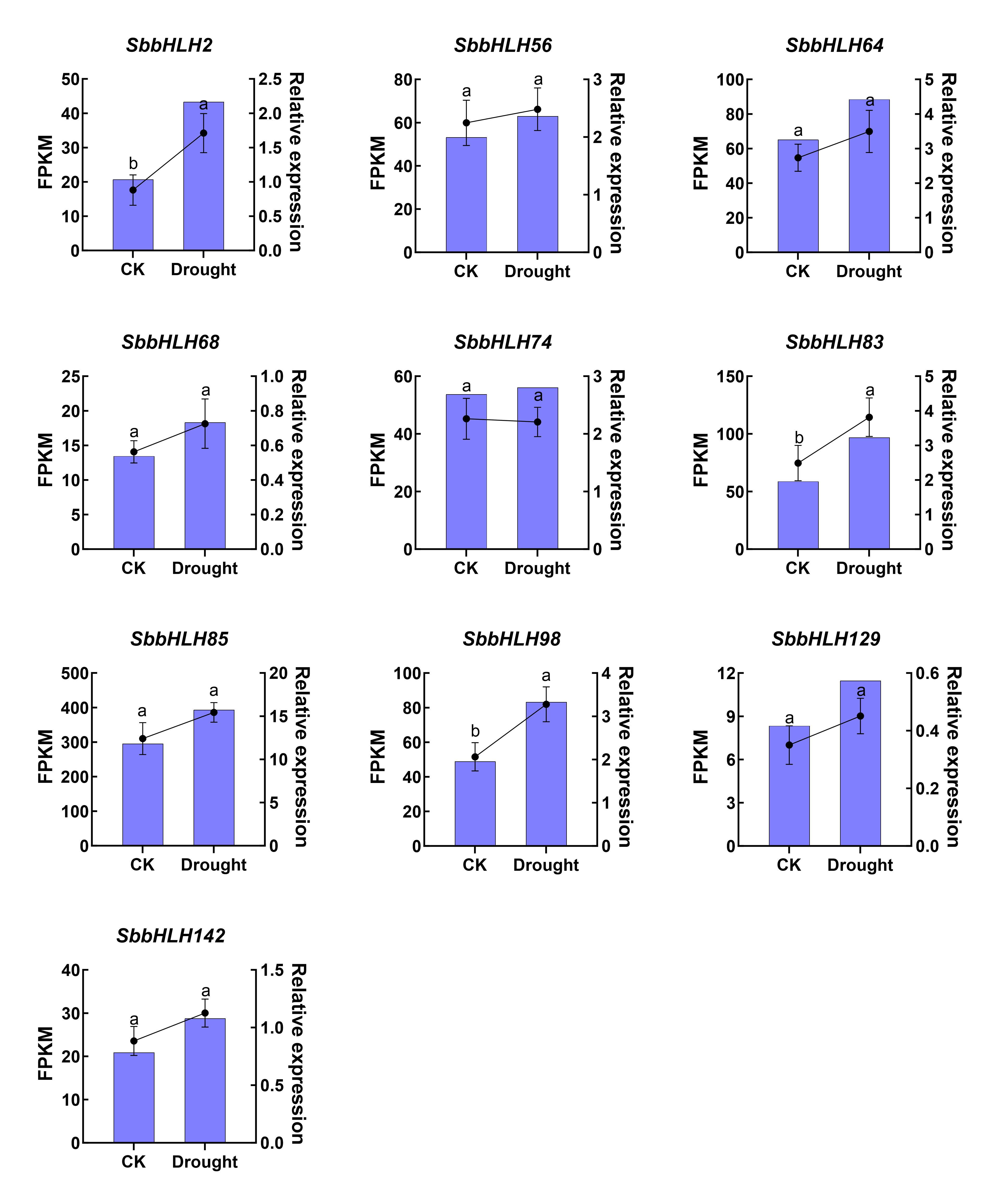


Fig. S4 Verification of transcriptome data of *S. baicalensis* under drought stress. The blue bar graph is the FPKM value of the transcriptome data, and the black line graph is the relative expression level of qRT-PCR, repeated 3 times. Statistical analysis was conducted with one-way analysis of variance, different lowercase letters indicate significant differences (*p*<0.05).


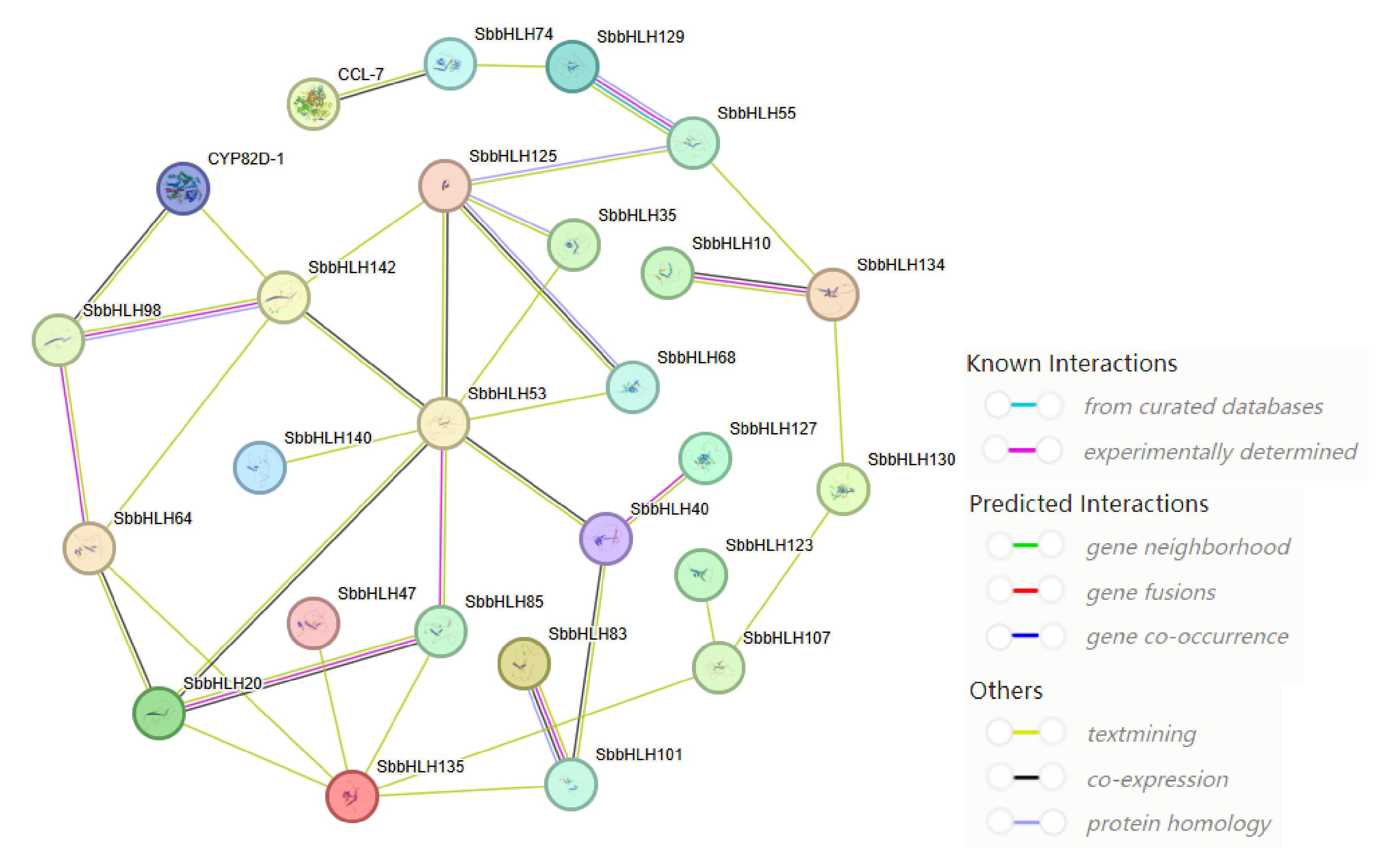


Fig. S5 Protein interaction network analysis. line color indicates the type of interaction evidence.
